# DRP3 and ELM1 are required for mitochondrial fission in the liverwort ***Marchantia polymorpha***

**DOI:** 10.1038/s41598-017-04886-0

**Published:** 2017-07-04

**Authors:** Nagisa Nagaoka, Akihiro Yamashita, Rina Kurisu, Yuta Watari, Fumiko Ishizuna, Nobuhissro Tsutsumi, Kimitsune Ishizaki, Takayuki Kohchi, Shin-ichi Arimura

**Affiliations:** 1 Graduate School of Agricultural and Life Sciences, The University of Tokyo, Tokyo, 113-8657 Japan; 2 Graduate School of Science, Kobe University, Kobe, 657-8501 Japan; 3 Graduate School of Biostudies, Kyoto University, Kyoto, 606-8502 Japan; 4 PRESTO, Japan Science and Technology Agency, Saitama, 332-0012 Japan

## Abstract

Mitochondria increase in number by the fission of existing mitochondria. Mitochondrial fission is needed to provide mitochondria to daughter cells during cell division. In *Arabidopsis thaliana*, four kinds of genes have been reported to be involved in mitochondrial fission. Two of them, *DRP3* (*dynamin-related protein3*) and *FIS1* (*FISSION1*), are well conserved in eukaryotes. The other two are plant-specific *ELM1* (*elongated mitochondria1*) and *PMD* (*peroxisomal and mitochondrial division*). To better understand the commonality and diversity of mitochondrial fission factors in land plants, we examined mitochondrial fission-related genes in a liverwort, *Marchantia polymorpha*. As a bryophyte, *M. polymorpha* has features distinct from those of the other land plant lineages. We found that *M. polymorpha* has single copies of homologues for *DRP3, FIS1* and *ELM1*, but does not appear to have a homologue of *PMD*. Citrine-fusion proteins with MpDRP3, MpFIS1 and MpELM1 were localized to mitochondria in *M. polymorpha*. Mp*DRP3*- and Mp*ELM1*-defective mutants grew slowly and had networked mitochondria, indicating that mitochondrial fission was blocked in the mutants, as expected. However, knockout of Mp*FIS1* did not affect growth or mitochondrial morphology. These results suggest that Mp*DRP3* and Mp*ELM1* but neither Mp*FIS1* nor *PMD* are needed for mitochondrial fission in *M. polymorpha*.

## Introduction

Almost all eukaryotes have mitochondria, organelles that are essential for energy production and supply, for the control of diverse metabolic pathways and for regulation of cell death. Mitochondria are dynamic organelles that move around and change their shape^1–4^. The morphology of mitochondria varies among species. In yeast and mammalian cells, mitochondria form networks that consists of tubules with multiple branch points^5, 6^. On the other hand, in plant cells, mitochondria are dispersed with many small spherical or peanut-shaped particles^1, 7^.

Mitochondria change their morphology depending on the life stage and growth conditions^5, 8, 9^. Both mitochondrial fission and fusion are needed for maintenance of mitochondrial morphology^10, 11^. Because mitochondria are not synthesized *de novo* but are created by the fission of existing mitochondria, mitochondrial fission is fundamental to the existence of eukaryotes^12, 13^. In yeast, a dynamin-related protein (Dnm1p) is recruited to mitochondrial fission sites where it forms an oligomer that constricts mitochondria to facilitate their fission^10, 14–16^. Dnm1p and its orthologues are well conserved in diverse eukaryotes including yeasts, algae, plants and mammals^13^. The *Arabidopsis thaliana* genome has two closely similar dynamin-related proteins, DRP3A and DRP3B, that are the functional orthologs of Dnm1p and that function in a redundant manner^7, 17–20^. In the *drp3a drp3b* double mutant, the mitochondria do not divide and form a massive, elongated network^20^.

In budding yeast, relocalization of Dnm1p depends on the mitochondrial outer membrane protein Fis1p and the cytosolic adaptor Mdv1p (and its paralog Caf4p), which can bind to both Dnm1p and Fis1p^11, 21^. In the *fis1* mutant, mitochondria were elongated and networked^22^. Although Fis1p is required in yeast, the roles of Fis1 homologues in mammalian cells are somewhat controversial. Overexpression of hFis1 of *Homo sapiens* leads to mitochondrial fragmentation, indicating that it has a role in regulating mitochondrial morphology^23, 24^. However, hFis1 doesn’t appear to be essential for localizing Drp1 to mitochondrial fission sites, as its role can be handled by two other outer-membrane proteins, Mff and MiD49/51^25–28^.

The Arabidopsis genome has two closely related homologues of *Fis1* (*FIS1A/BIGYIN* and *FIS1B*). FIS1A and FIS1B are reported to target to mitochondria, peroxisomes and chloroplasts, and were shown to facilitate mitochondrial fission^29–31^. BLAST searches of the Arabidopsis genome (https://www.arabidopsis.org/Blast/index.jsp) did not reveal any homologues of other mitochondrial fission factors (Mdv1p/Caf4p, Mff or MiD49/51). The closest matches had e-values > 1e-4, indicating very weak matches. On the other hand, it has two plant-specific fission factors, elongated mitochondria1 (*ELM1*) and peroxisomal and mitochondrial division factors (*PMD*s). *ELM1* was identified as the gene responsible for the elongated mitochondria mutant phenotype in Arabidopsis. In wild-type Arabidopsis, ELM1 surrounds mitochondria and interacts with both DRP3A and DRP3B^32^. These results suggest that ELM1 is required for relocalization of DRP3A from the cytosol to mitochondrial fission sites. Elongated mitochondria are also observed in the *pmd1* mutant, but PMD1 and its paralog PMD2 do not physically interact with DRP3 or FIS1, suggesting that PMD1 facilitates mitochondrial proliferation in a DRP3/FIS1-independent manner^33^.

Recent molecular phylogenetic analyses of land plants agreed the basal position bryophytes, encompassing liverworts, mosses and hornworts, to vascular plants, and liverworts are considered as one of the earliest diverging distant land plant lineages^34–36^. Therefore, liverworts are could be a key clade to our understanding of the commonalities and differences of mitochondrial fission factors among land plants. Here, we used reverse genetics to study candidate genes for mitochondrial fission in the liverwort *Marchantia polymorpha*. Marchantia is emerging as an experimental model organism, because it has little genetic redundancy in many cases and is well suited for various strategies for molecular genetics, such as introduction of reporter constructs, gene silencing and targeted gene modification^37–40^. Our results demonstrate that mitochondrial fission in a liverwort relies on some factors used in vascular plants (DRP3 and ELM1) but does not seem to rely on other factors (FIS1 and PMDs).

## Results

### The Marchantia genome has single copies of homologues of *DRP3*, *FIS1* and *ELM1*

**Figure 1:**
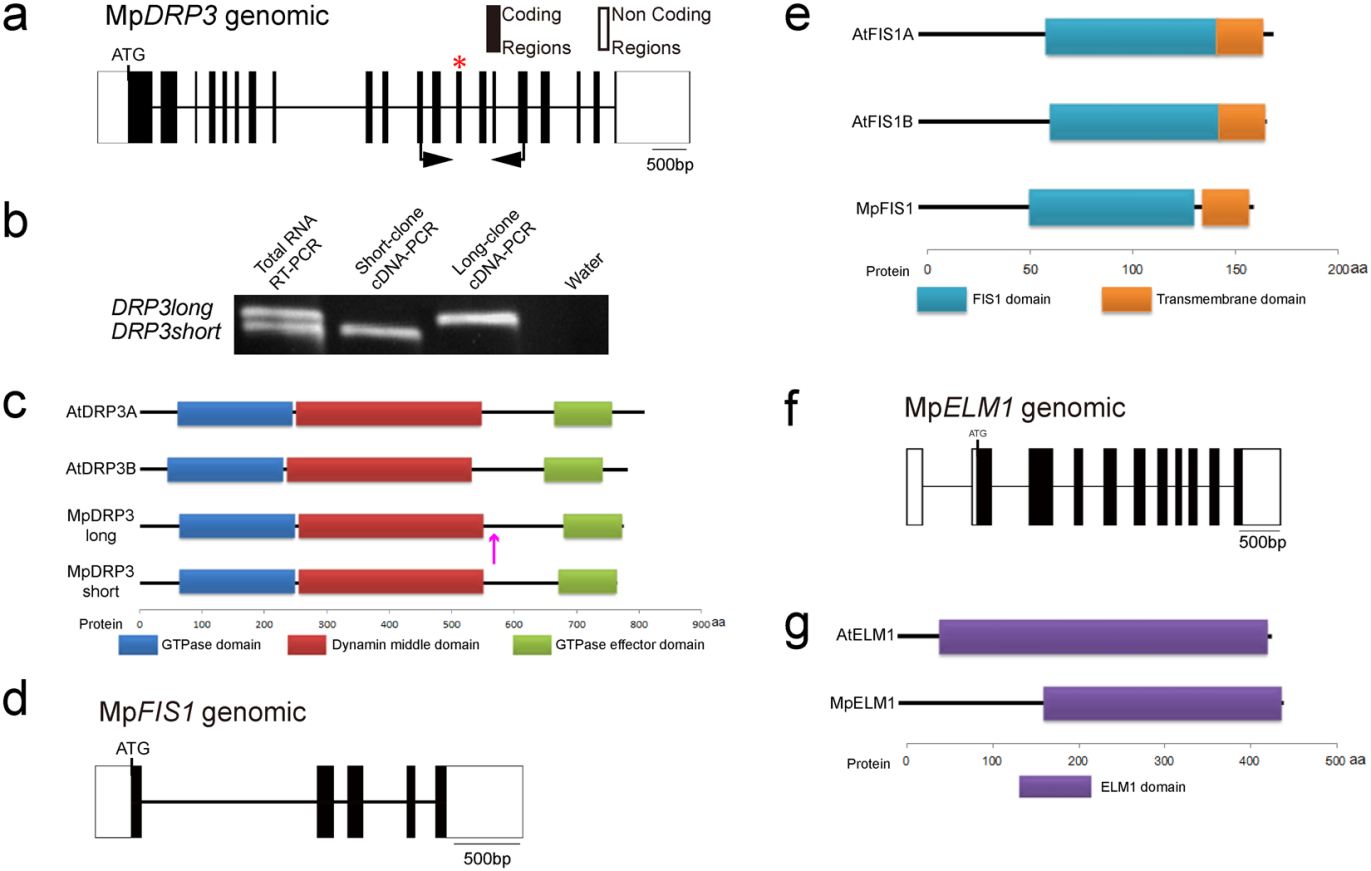
Genes and predicted proteins information of MpDRP3, MpFIS1 and MpELM1. (**a**) Genomic structure of Mp*DRP3*. The splice site variant was identified at exon 13 (denoted by the asterisk). Closed arrows indicate the positions of primers used in (**b**). (**b**) RT-PCR analysis of RNA extracted from the detached thallus. A full-length gel image is presented in Figure S6a. (**c**) Domain structures of AtDRP3A, AtDRP3B, MpDRP3s and MpDRP3l. Each domain is identified and depicted by using the pfam program (http://pfam.sanger.ac.uk/). The magenta colored arrow indicates the splice site. (**d**) Genomic structure of Mp*FIS1*. (**e**) Domain structures of AtFIS1A, AtFIS1B and MpFIS1. Each domain is identified and depicted by using the pfam program. (**f**) Genomic structure of Mp*ELM1*. (**g**) Domain structures of AtELM1 and MpELM1. Each domain is identified and depicted by using the pfam program.

For searches of the *M. polymorpha* genome, we used the JGI *M. polymorpha* EST and genome databases ver. 3.1 (https://phytozome.jgi.doe.gov/pz/portal.html). BLASTx searches of the JGI *M. polymorpha* EST and genome databases for homologues of *DRP3*, *FIS1*, *ELM1* and *PMD* revealed no *PMD* homologue (e-value of closest match > 1e-4), but did reveal single copies of homologues of *DRP3* (0.0 or 6e-71), *FIS1* (5e-35) and *ELM1* (1e-138). We call these homologues Mp*DRP3* (Mapoly0069s0084), Mp*FIS1* (Mapoly0147s0019) and Mp*ELM1* (Mapoly0038s0050), respectively. Two different mRNAs of Mp*DRP3* were found and appear to be transcribed from a single gene (Figs 1a–c and S1a). The predicted splicing variants, Mp*DRP3s* (short) and Mp*DRP3l* (long), differed by 27 bp in exon 13. RT-PCR analysis revealed that the mRNA expression levels of Mp*DRP3s* and Mp*DRP3l* in the thallus were similar (Fig. 1b). The amino acid sequences of MpDRP3s and MpDRP3l are highly similar to their Arabidopsis and Physcomitrella homologues (Fig. S1a). MpDRP3s had 62.4% identity to AtDRP3A and 79.7% identity to PpDRP3. The alternative splice site is not conserved in Arabidopsis and Physcomitrella (Fig. S1a, magenta). In addition, all five genes have predicted GTPase, dynamin middle and GTPase effector domains (Fig. 1c). Similarly, MpFIS1 (Fig. 1d) and MpELM1 (Fig. 1f) had high similarities in length, amino-acid sequences (Fig. S1b and c) and domain structures (Fig. 1e and g) to Arabidopsis and Physcomitrella counterparts, respectively.

### MpDRP3, MpFIS1 and MpELM1 were localized to mitochondria

**Figure 2:**
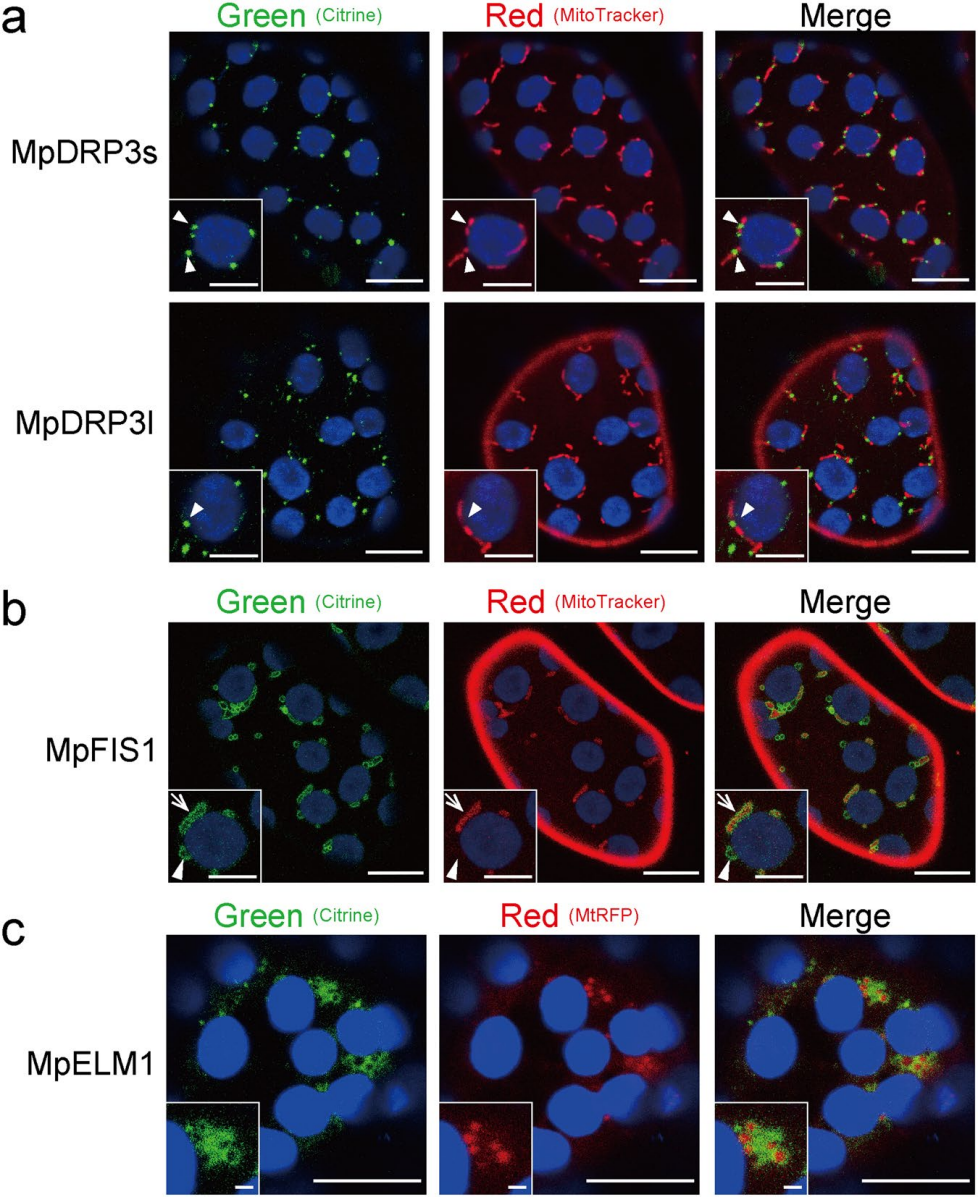
Intracellular localization of MpDRP3, MpFIS1 and MpELM1 in Marchantia thallus. (**a**) CLSM images of the epidermal cell expressing Citrine-MpDRP3s (upper panel) or Citrine-MpDRP3l (lower panel) and stained with MitoTracker (Bars = 10 µm). Magnified images are shown in insets (Bars = 5 µm). The arrowheads indicate Citrine signals localized to the constriction sites of mitochondria. (**b**) CLSM image of the epidermal cell expressing Citrine-MpFIS1 and stained with MitoTracker (Bars = 10 µm). Magnified images are shown in insets (Bars = 5 µm). The arrow indicates a mitochondrion and the arrowhead indicates a body not mitochondria. (**c**) Fluorescence image of the epidermal cell transiently transformed with Citrine-MpELM1 and MtRFP by particle bombardment (Bars = 10 µm). Magnified images are shown in insets (Bars = 1 µm). Blue signals were chlorophyll autofluorescence.

To examine the subcellular localization of MpDRP3s and MpDRP3l, we observed a fluorescent marker Citrine-fused proteins (Citrine-MpDRP3s and Citrine-MpDRP3l) with a confocal laser scanning microscope (CLSM)(Fig. 2a). The fusion genes were given an N-terminal Citrine tag and placed under the control of the CaMV 35 S promoter. In transgenic plants expressing these proteins and stained with MitoTracker, a part of the Citrine-MpDRP3s and Citrine-MpDRP3l signals localized to the constriction sites of mitochondria (Fig. 2a, arrowheads). Other signals were mainly localized to the tips of mitochondria, while some remained in the cytosol. To better see the localizations of MpDRP3s or MpDRP3l, we prepared another reporter protein: RFP fused to the N-terminus of MpDRP3s or MpDRP3l, and observed cells expressing both Citrine-MpDRP3s and RFP-MpDRP3l (or Citrine-MpDRP3l and RFP-MpDRP3s) (Fig. S2a). MpDRP3s and MpDRP3l signals overlapped in each combination of plasmids, suggesting that they took part in the same fission machinery (Fig. S2b). Next, the DNA fragment *35Spro:Citrine:MpFIS1* was introduced into Marchantia to identify the localization of MpFIS1 within the cells. In transgenic plants expressing Citrine-MpFIS1 and stained with MitoTracker, almost all Citrine fluorescence was in the form of green oval-rings. Many of the rings surrounded the MitoTracker fluorescence of mitochondria (Fig. 2b, arrow) while some did not contain the red signals (Fig. 2b, arrowhead). Transient expression of Citrine-MpFIS1 and RFP fused to the peroxisomal targeting signal 2 (PTS2-RFP) showed that a part of Citrine-MpFIS1 colocalized with PTS2-RFP (Fig. S3). In addition, Marchantia thalli were transformed transiently with MpELM1-Citrine and mitochondrial-targeted RFP (MtRFP) by particle bombardment. Although the mitochondria appeared to be enlarged and aggregated, at least some of the MpELM1-Citrine surrounded the RFP fluorescence of mitochondria (Fig. 2c).

### Mp*elm1* and Mp*drp3* (but not Mp*fis1*) showed significant growth defects

**Figure 3:**
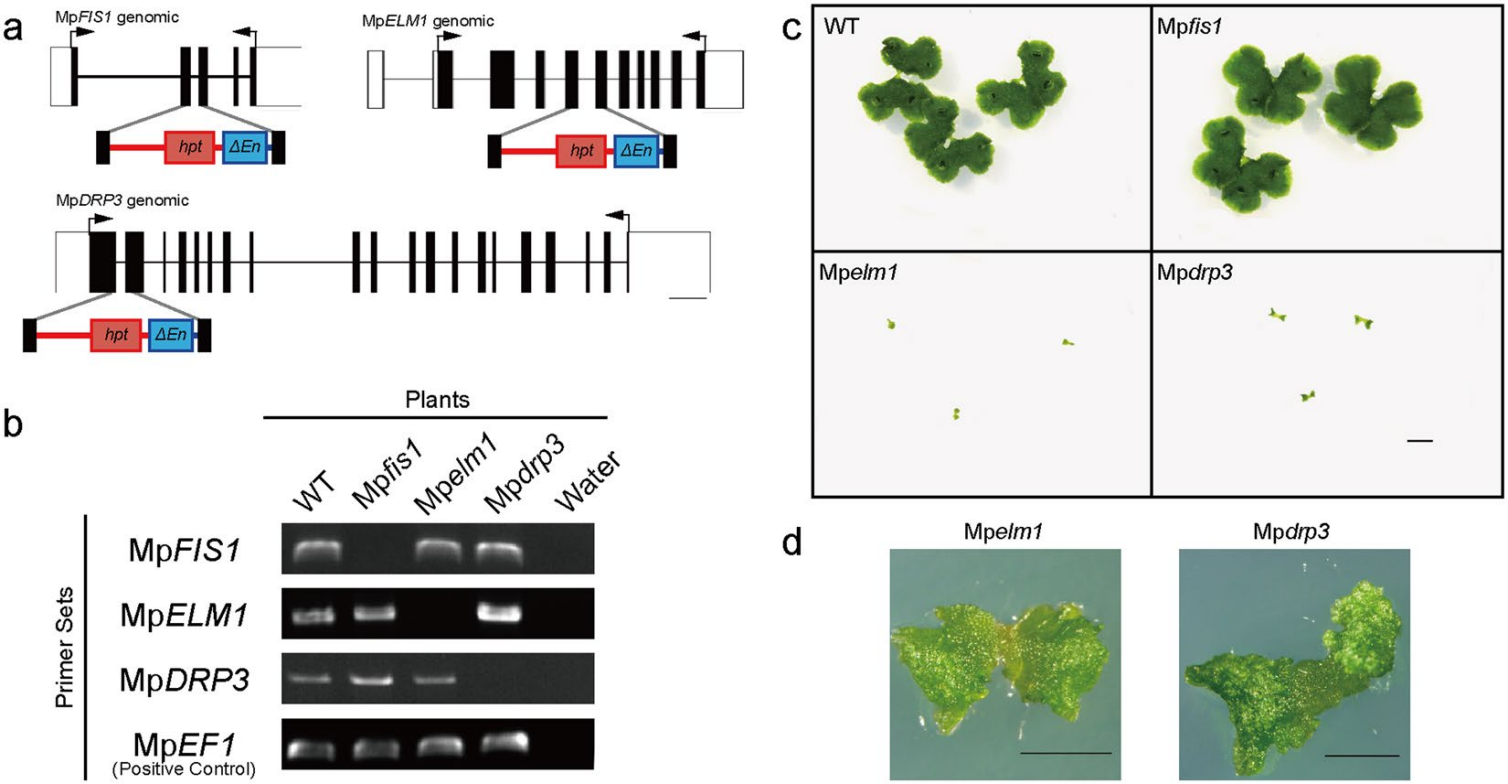
Generation of Mp*fis1*, Mp*elm1* and Mp*drp3* plants by homologous recombination and plant phenotypes of the mutants. (**a**) Schematic representation of the genomic structures of the Mp*FIS1*, Mp*ELM1* and Mp*DRP3*, the disrupting-constructs and their targeting loci. Each primer pair for RT-PCR is indicated by arrows. (**b**) RT-PCR for Mp*FIS1*, Mp*ELM1* and Mp*DRP3* mRNA in wild-type (WT), Mp*fis1*, Mp*elm1* and Mp*drp3* plants. Mp*EF1* (Elongation Factor 1 alpha) was used as an internal control. Full-length gel images are presented in Figure S6b. (**c**) Photographs of 2-weeks-old plants of wild-type (WT), Mp*fis1*, Mp*elm1* and Mp*drp3*. All plants shown in this image were grown on a single plate. Bar = 5 mm. (**d**) Magnified images of distorted thalli of Mp*elm1* and Mp*drp3* by using a stereoscopic microscope. Bars = 1 mm.

To clarify the role of each gene in mitochondrial fission and plant growth, we made Mp*FIS1*, Mp*ELM1* and Mp*DRP3* knockout mutants (named Mp*fis1*, Mp*elm1* and Mp*drp3*, respectively) by homologous recombination (HR)-mediated gene targeting (Fig. 3a)^38^. The knockouts were confirmed by RT-PCR (Fig. 3b). The wild-type plants grew to an area of about 200 mm^2^ in 2-weeks from a gemma (Fig. 3c). Compared with the wild type, Mp*fis1* did not have any noticeable developmental or growth defects (Fig. 3c). In contrast, Mp*elm1* and Mp*drp3* showed similar growth retardation, i.e., their areas grew to less than 10 mm^2^ in 2 weeks from a gemma. In addition, Mp*elm1* and Mp*drp3* showed distorted thalli phenotypes (Fig. 3d), and they began to wither before reaching the wild-type size. However, these mutants were able to complete their asexual life cycle, i.e., they generated asexual propagules called gemmae, which develop into adult gametophytes.

### Mp*elm1* and Mp*drp3* (but not Mp*fis1*) showed severe morphological phenotypes of mitochondria

**Figure 4:**
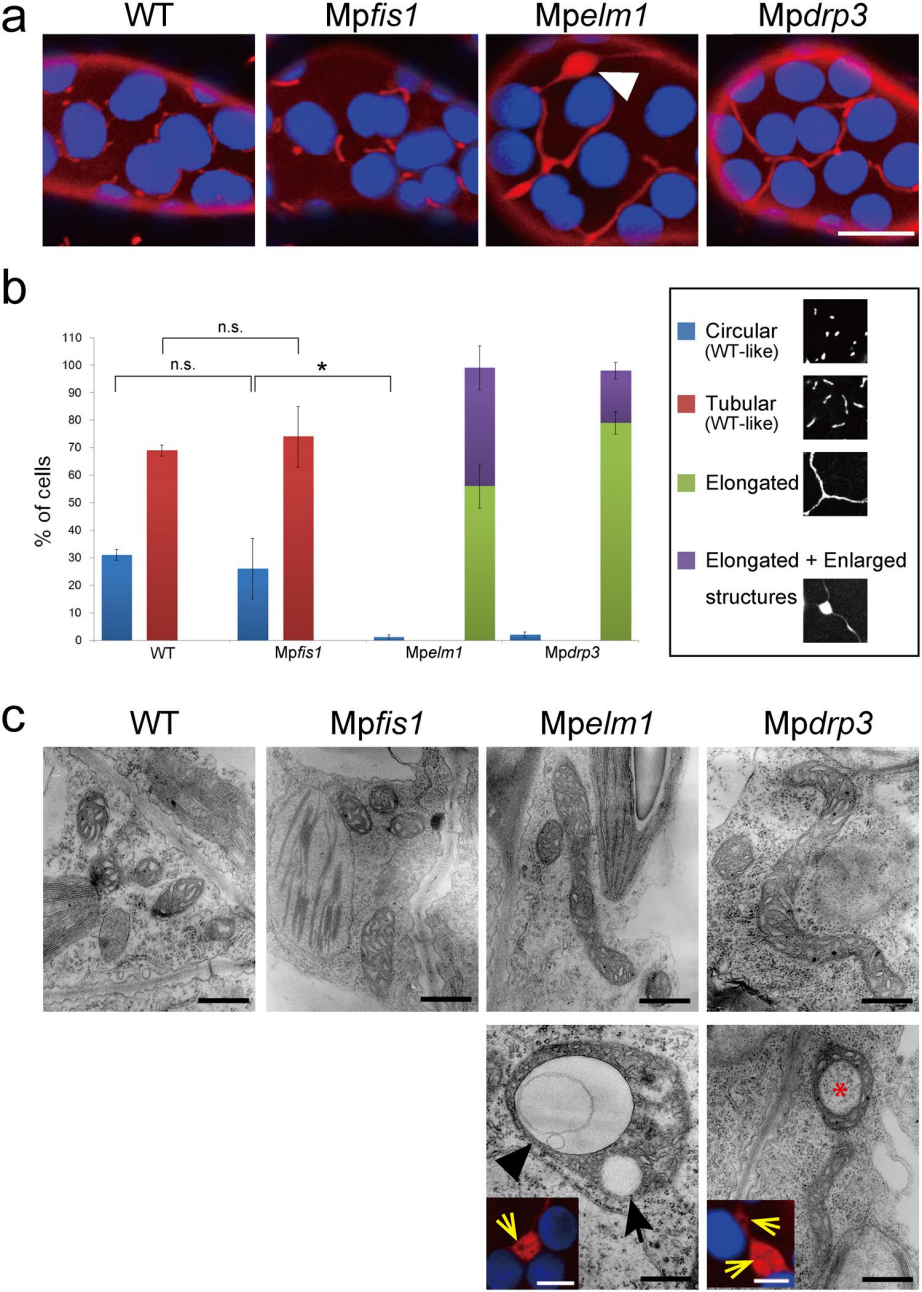
Mitochondrial phenotypes of the mutants. (**a**) CLSM image of the epidermal cell in wild-type and each mutants stained with MitoTracker. The arrowhead indicates an enlarged structure of mitochondria. Blue signals are chlorophyll autofluorescence. Bar = 10 µm. (**b**) Scoring of mitochondrial morphologies. Each cell was scored into one of four morphological categories. Representative images of each categories are shown in right panels. Data are means ± s.e.m. from three independent experiments. *P < 0.05; n.s., not significant; one-way ANOVA with Tukey’s multiple-comparison test. (**c**) Transmission electron micrographs of mitochondria in wild-type and each mutants. Lower panels are magnified images of enlarged structures in Mp*elm1* and Mp*drp3*. The black arrow indicates a vacuolated structure, the black arrowhead indicates a vesicle-like structure and the asterisk indicates the cytosol surrounded by mitochondria (Bars = 500 nm). Fluorescent images of enlarged structures stained with MitoTracker are shown in insets. The yellow arrows indicate the mitochondrial region which has little MitoTracker signals (Bars = 5 µm).

We then examined whether knockout of those genes affects mitochondrial morphology. In wild-type and Mp*fis1* cells, most mitochondria were observed as particles or narrow (0.5 to 1 µm) rod-like structures (Fig. 4a). Citrine-MpDRP3s and MpELM1-Citrine in Mp*fis1* localized to mitochondria (Fig. S4a and S4b respectively), as they did in the wild type (Fig. 2a and c, respectively). By contrast, massively elongated and interconnected mitochondria were observed in Mp*elm1* and Mp*drp3* with some lump-like structures (Fig. 4a, arrowhead). To compare the mutants quantitatively by their mitochondrial morphology, we used a morphology scoring assay in which each cell was categorized as having one of four types of mitochondria: 1) circular, 2) tubular, 3) elongated and 4) elongated + enlarged (i.e., mitochondria interconnected with some lump-like structures). Following previous studies^10, 15, 26^, we compared the phenotypes of mitochondria in similar volumes and the same cell numbers using a cell-based assay, rather than a mitochondrion-based assay. Although there were no “Elongated” cells in the wild-type or Mp*fis1*, most of the cells in Mp*elm1* and Mp*drp3* were categorized as “Elongated” (Fig. 4b), indicating that mitochondrial fission was blocked in Mp*elm1* and Mp*drp3*, but not in Mp*fis1*.

As with the fluorescence microscopy, transmission electron microscopy showed that most of mitochondria in Mp*elm1* and Mp*drp3* were much longer than those in the wild-type and Mp*fis1* (Fig. 4c). In addition, enlarged structures of mitochondria included vesicle-like structures (black arrowhead) and vacuolated structures (black arrow). These structures might be similar to the dark areas inside mitochondrial red bodies (shown in the insets of Fig. 4c (yellow arrows)), which were not stained with MitoTracker. Although Mp*elm1* and Mp*drp3* had enlarged mitochondria, their cristae were similar to those in the wild-type, and they maintained their electrochemical potential because the MitoTracker that we used (Orange CMTMRos) cannot accumulate in mitochondria that do not maintain a membrane potential.

### Transient expression of Mp*DRP3* and Mp*ELM1* in the mutants complemented the morphological phenotypes of mitochondria

**Figure 5:**
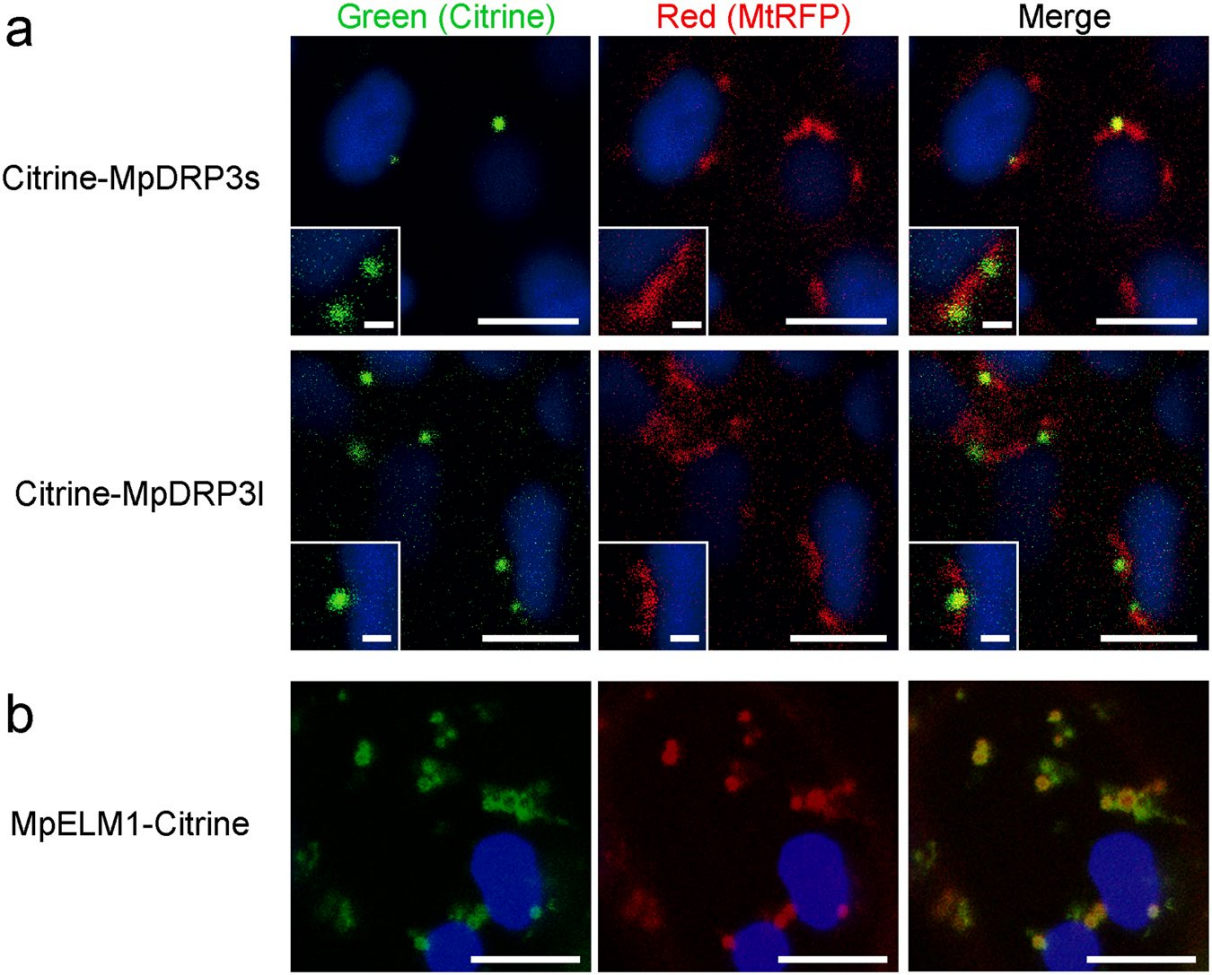
Complementation analysis of mutants lacking MpDRP3 or MpELM1. (**a**) CLSM observations of either Citrine-MpDRP3s or Citrine-MpDRP3l and MtRFP in Mp*drp3* by using the particle bombardment (Bars = 5 µm). Those were transiently expressed under the control of the CaMV 35 S promoter. Magnified images are shown in insets (Bars = 1 µm). (**b**) CLSM Images of MpELM1-Citrine and MtRFP in Mp*elm1* (Bars = 5 µm). Blue signals were chlorophyll autofluorescence.

Because MpDRP3s and MpDRP3l had similar subcellular localizations, we examined whether both of them were functional. To test this, we examined the ability of the MpDRP3s and MpDRP3l to complement the defective mitochondrial networking phenotype of Marchantia lacking MpDRP3. When Mp*drp3* was transformed transiently with either Citrine-MpDRP3s or Citrine-MpDRP3l by particle bombardment, the elongated mitochondrial morphology reverted to a wild-type-like morphology. Similarly, Mp*elm1* cells with MpELM1-Citrine have discrete complemented mitochondria (Fig. 5b).

## Discussion

In this work, three of four candidate genes for mitochondrial fission (Mp*DRP3*, Mp*FIS1* and Mp*ELM1*) were found in the genomic database of *Marchantia polymorpha* (Fig. 1). The finding that these three genes (but not *PMD*) are conserved in Arabidopsis and Marchantia, indicate that Mp*DRP3*, Mp*FIS1* and Mp*ELM1* have significant roles in land plants. The Arabidopsis genome has two copies of *DRP3*-, *FIS1*- and *ELM1-*type genes^13^. In contrast, Marchantia has single copies of these genes, which makes it a good model for revealing the mechanism of mitochondrial fission in land plants.

Mp*DRP3* exists in two alternative splicing isoforms (Mp*DRP3s* and Mp*DRP3l*) that differ by 27 bp in exon 13 (Fig. 1). The mRNA expression levels of Mp*DRP3s* and Mp*DRP3l* in the thallus were similar (Fig. 1b). Furthermore, the mitochondrial morphology in Mp*drp3* was rescued by the expression of either Citrine-MpDRP3s or Citrine-MpDRP3l transiently (Fig. 5a). Together, these findings suggest that MpDRP3s and MpDRP3l are functionally redundant, at least in the observed Marchantia thallus cells.

Because little or no difference was observed in plant growth (Fig. 3c) or mitochondrial morphology (Fig. 4b) between Mp*fis1* and the wild-type, MpFIS1 does not appear to have a major role in mitochondrial fission. The mitochondrial localizations of Citrine-MpDRP3s and MpELM1-Citrine in Mp*fis1* mutants in Fig. S4a and S4b suggest that MpFIS1, unlike yeast FIS1, is not required for the localization of these mitochondrial fission proteins from the cytosol. Citrine-MpFIS1, which was detected on the mitochondrial surface (Fig. 2b), probably binds by the C-terminal transmembrane domain of MpFIS1, as this domain is crucial for mitochondrial targeting in other eukaryotes^11, 23, 24^. Furthermore, Citrine-MpFIS1 was localized to ring-shaped bodies (Fig. 2b, left panel, arrowhead), which seems to be peroxisomes (Fig. S3). Although FIS1 also localizes to peroxisomes and is involved in peroxisomal fission in Arabidopsis^30, 31, 41, 42^, the role of MpFIS1 in peroxisomal fission remains unclear.

Moreover, phenotypic analyses of plant growth (Fig. 3c) and mitochondrial morphology (Fig. 4b) suggested that not only MpDRP3 but also MpELM1 play crucial roles in mitochondrial fission. In Arabidopsis, *drp3a drp3b* double mutants show a severe growth-defect with network-shaped mitochondria^20^, but *elm1* mutants do not show growth defects^32^. This is possibly because the latter mutants have residual mitochondrial fission activity, attributed to another *ELM1* paralogue (At5g06180) in the Arabidopsis genome^32^. By contrast, the Marchantia genome has a single copy of *ELM1*, so that there would be no residual mitochondrial fission activity in Mp*elm1* mutants.

**Figure 6:**
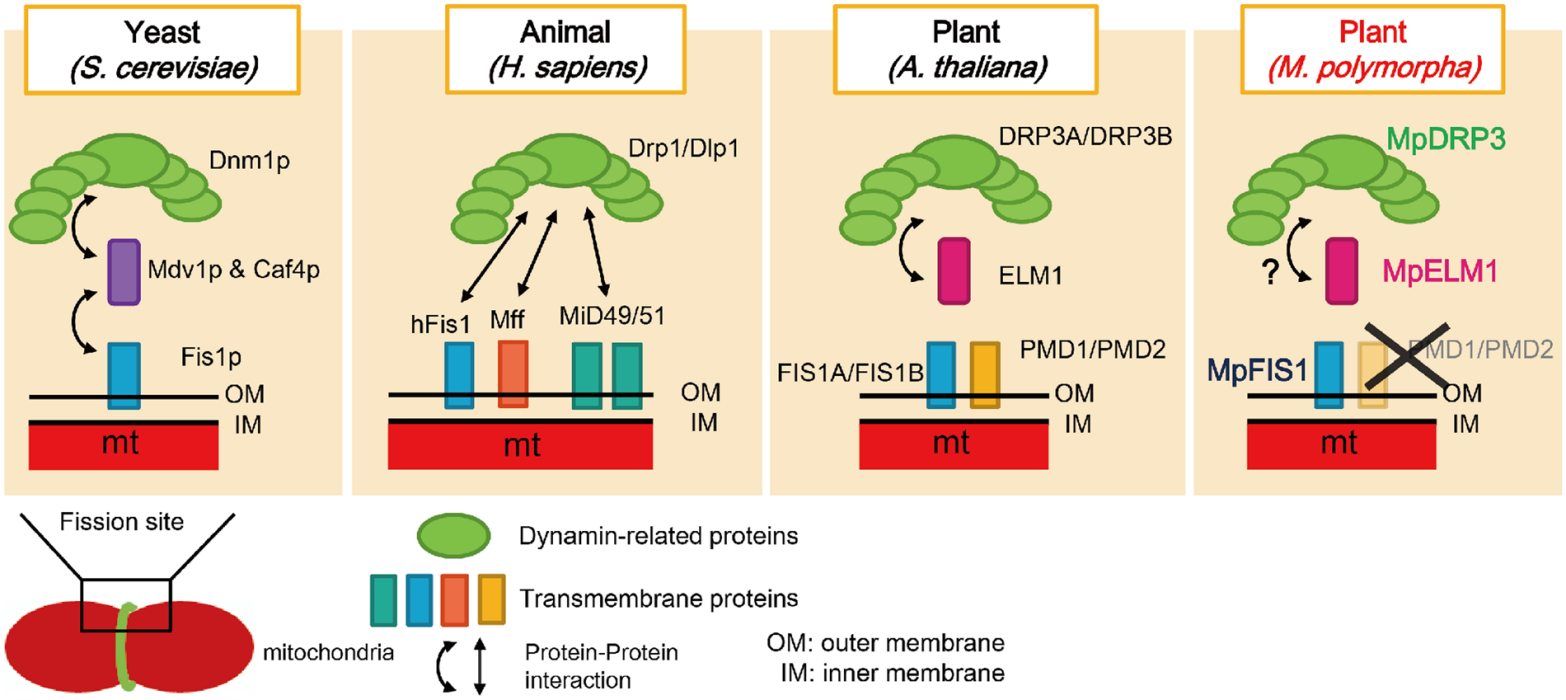
Comparison of mitochondrial fission factors in yeast, an animal and plants. These are current models of mitochondrial fission in *S. cerevisiae*, *H. sapiens*, *A. thaliana* and *M. polymorpha*.

The phenotypes in Mp*elm1* strongly resemble those in Mp*drp3* (Figs 3c and 4a), which raises the possibility that MpELM1 is involved in an MpDRP3-dependent pathway. In other words, MpDRP3 may require MpELM1, in the same way that Dnm1p requires the cytosolic adaptor Mdv1p/Caf4p, which localizes Dnm1p to the mitochondrial fission sites in budding yeast^11, 21^ (Fig. 6, left). It is possible that MpDRP3 interacts with MpELM1, since DRP3A and DRP3B interact with ELM1 for their localization to mitochondria in Arabidopsis (two-way arrows in Fig. 6)^32^. Further biochemical studies in Marchantia are needed to test this model.

Dynamin-related proteins (green shapes in Fig. 6) are universally conserved in eukaryotes, including yeasts, humans and vascular plants^7, 14, 43, 44^. The present results show that this is also the case in Marchantia. Indeed, MpDRP3 had high identities with DRP3 orthologues from *A. thaliana* and *P. patens* (Fig. 1c), suggesting that dynamin-related proteins are also crucial in bryophytes. Although the amino acid sequences of Fis1 homologues (blue bars in Fig. 6) are conserved across diverse eukaryotes and Fis1 plays an integral part for mitochondrial fission in yeast, it remains unclear whether Fis1 has a role in mitochondrial fission in mammals^26^. Considering that mammalian membrane-anchored proteins (Mff and MiD49/51) act as mitochondrial adaptors for Drp1 in place of (or with) Fis1^25–28^, it is possible that *M. polymorpha* has other mitochondrial fission factor(s) on the surface of mitochondria. Because MpFIS1 does not appear to play a role in mitochondrial fission (Fig. 4b), it probably has some other function. This raises the possibility that Fis1s in other eukaryotes might also have some other common function.

The Marchantia genome does not seem to have a *PMD* homologue. A search of the databases shows that homologues of *PMD*s are found only in Brassicaceae, a family of vascular plants, and related plants, suggesting that PMD is a mitochondrial fission factor specific to these plants. On the other hand, ELM1-like proteins are evolutionarily conserved across land plants^32^, but they are not present in the genomes of chlorophytes (*Chlamydomonas reinhardtii*, *Volvox carteri* and *Chlorella vulgaris*) or a rhodophyte (*Cyanidioschyzon merolae*). The present results show that the amino acid sequence and the function of ELM1 in the vascular plant Arabidopsis are conserved in a bryophyte, indicating that the ancestor of land plants acquired ELM1 as a mitochondrial fission factor.

## Experimental procedures

### Plant materials and growth conditions

Male, accession Takaragaike-1 (called Tak-1), *M. polymorpha* were asexually maintained and propagated through gemmae growth at 22 °C by using half-strength Gamborg’s B5 medium containing 1% agar under 16-h of diurnal light or continuous white light (50–100 µmol m^−2^sec^−1^)^37, 45^.

### Plasmid constructions

Gateway cloning technology (Life Technologies) was used to construct plasmids. cDNA was amplified by PCR with the following primers (Table S1). The amplified DNA fragments were mixed with an entry vector, pENTR/D-TOPO, and the TOPO reaction were performed according to the manufacturer’s instructions (Life Technologies). In addition, the stop codon of pENTR-Mp*ELM1* was deleted by using Site-Directed Mutagenesis to add the fluorescent protein to their C-terminus. The resulting plasmids were mixed and reacted with an LR clonase with destination vectors, p2RGW7^46^, pMpGWB105^47^ or pMpGWB106^47^, to construct binary vectors containing the CaMV 35 S promoter, fluorescent genes and a NOS terminator.

### RT-PCR analysis

Total RNA was extracted from sections of thallus. RNA isolation was carried out using the RNeasy Plant Mini Kit (QIAGEN), according to manufacturer’s instructions. First strand cDNA synthesis was carried out starting from 1 µg of total RNA by using SuperScript III Reverse Transcriptase (Invitrogen) and the oligo(dT) primers for reverse transcription. To detect the splicing variants and transcriptional products, RT-PCR analyses were performed using appropriate primers (Table S1). The expression levels of each gene were normalized to the expression level of the Mp*EF1*.

### Putative amino-acid sequence and domain structures

Sequence comparisons were performed by using the CLUSTAL W multiple alignment program and GeneDoc Program between sequences of Marchantia, Arabidopsis and Physcomitrella in NCBI database^48^. To analyse domain structures, we used Conserved Domain Search Service by NCBI (http://www.ncbi.nlm.nih.gov/). SOSUI and TMHMM transmembrane domain searches predicted transmembrane domain in the MpELM1 sequence^49, 50^.

### Agrobacterium-mediated transformation

Transgenic plants were generated with AgarTrap (Agar-utilized Transformation with Pouring Solutions) as described previously^40^. Transformants were selected on plates containing hygromycin B (10 µg ml^−1^) and claforan (100 µg ml^−1^) and cultivated for approximately 1 month to obtain gemmae. Then, a gemma was picked to establish an isogenic line from the respective transgenic lines, as a gemma is developed from a single cell^39^.

### Particle bombardment

Plasmids for visualizing mitochondrial fission factors were introduced via particle bombardment, by using a helium-driven particle accelerator (Bio-Rad, PDS/1000), with all basic adjustments set according to the manufacturer’s recommendations. The bombardment parameters applied were as follows: bombardment pressure, 7.6 × 10^4^ hPa; gold particles of 1.6 µm in diameter; 12 cm between the macrocarrier and plants; decompression vacuum, 915 hPa. After bombardment, plants were incubated for 2-days and observed by using CLSM.

### MitoTracker Orange staining

Small sections (2–10 mm^2^) were cut out of the Marchantia thallus with a sharp razor blade, stained with 0.5 µM MitoTracker Orange CMTMRos (Life Technologies) for about 20 minutes, and washed three times with distilled water.

### Microscopic observations and image analysis

Microscopic observations were performed by using a transmission electron microscope (JEOL, JEM-1010), a stereoscopic microscope (Leica, M125), a fluorescence microscope (Nikon, ECLIPSE Ti) and a confocal laser scanning microscope system (Nikon, C1Si). For the electron microscopic analysis, the pre-fixation was performed with phosphate buffer (50 mM) containing 4% paraformaldehyde and 2% glutaraldehyde. The pre-fixed samples were then post-fixed with 2% osmium tetroxide. The specimens were dehydrated in a graded ethanol series and embedded in Spurr resin (Spurr Low Viscosity Embedding Kit, Polysciences). Ultrathin sections (70 nm) were prepared and stained with 2% uranyl acetate and lead solution.

In fluorescence imaging, a 488-nm Ar/Kr laser was used for the excitation of Citrine. A 561-nm diode laser was also used for RFP, MitoTracker Orange and autofluorescence from chloroplasts. Emission signals were detected using a 515/30-nm filter for Citrine, a 590/70-nm filter for RFP and MitoTracker Orange and a 650-nm filter for autofluorescence. All of the data were processed using Adobe Photoshop CS4 Extended 11.0 (Adobe Systems).

### Targeted gene knockout

To generate the targeting vectors, pJHY-TMp1 was used^38^. The 5′ and 3′ homology arms were amplified with the appropriate primer pairs, and the PCR products were cloned into the *Asc*I and *Pac*I sites of pJHY-TMp1, respectively (Tables S1 and S2). Introduction of the targeting construct into *M. polymorpha* and selection of knockout lines were performed as previously described^37, 38^.

### Quantification of mitochondrial morphology

One experimenter stained the mutant and wild-type plants with MitoTracker Orange, and obtained images of 50 epidermal cells with CLSM in each experiment for three replicates. A second experimenter, blinded to the preparation of the images, categorized each cell into four groups (circular, tubular, elongated and elongated + enlarged structures) based on mitochondrial morphologies. All statistical testing was performed with one-way analysis of variance (ANOVA) followed by Tukey’s multiple-comparison test. *P* < 0.05 was considered to be statistically significant; n.s., not significant.

### Accession numbers

The sequences of the Mp*EF1* (*Elongation Factor 1 alpha*), Mp*DRP3*, Mp*ELM1* and Mp*FIS1* genes are available in DDBJ under the following accession numbers: Mp*EF1* (KJ146970); Mp*DRP3* (LC209090); Mp*ELM1* (LC209091); Mp*FIS1* (LC209092).

## Electronic supplementary material

Supplemental Figures
