# Supplementary material for: DRP3 and ELM1 are required for mitochondrial fission in the liverwort Marchantia polymorpha
Source: Sci Rep. 2017 Jul 4;7:4600. doi: 10.1038/s41598-017-04886-0 (PMC5496855; doi:10.1038/s41598-017-04886-0)
Supplement: Supplementary file 1 — Supplemental Figures [file 41598_2017_4886_MOESM1_ESM.pdf]

DRP3 and ELM1 are required for mitochondrial fission in the liverwort *Marchantia polymorpha*

Nagisa Nagaoka, Akihiro Yamashita, Rina Kurisu, Yuta Watari, Fumiko Ishizuna, Nobuhiro Tsutsumi, Kimitsune Ishizaki, Takayuki Kohchi & Shin-ichi Arimura

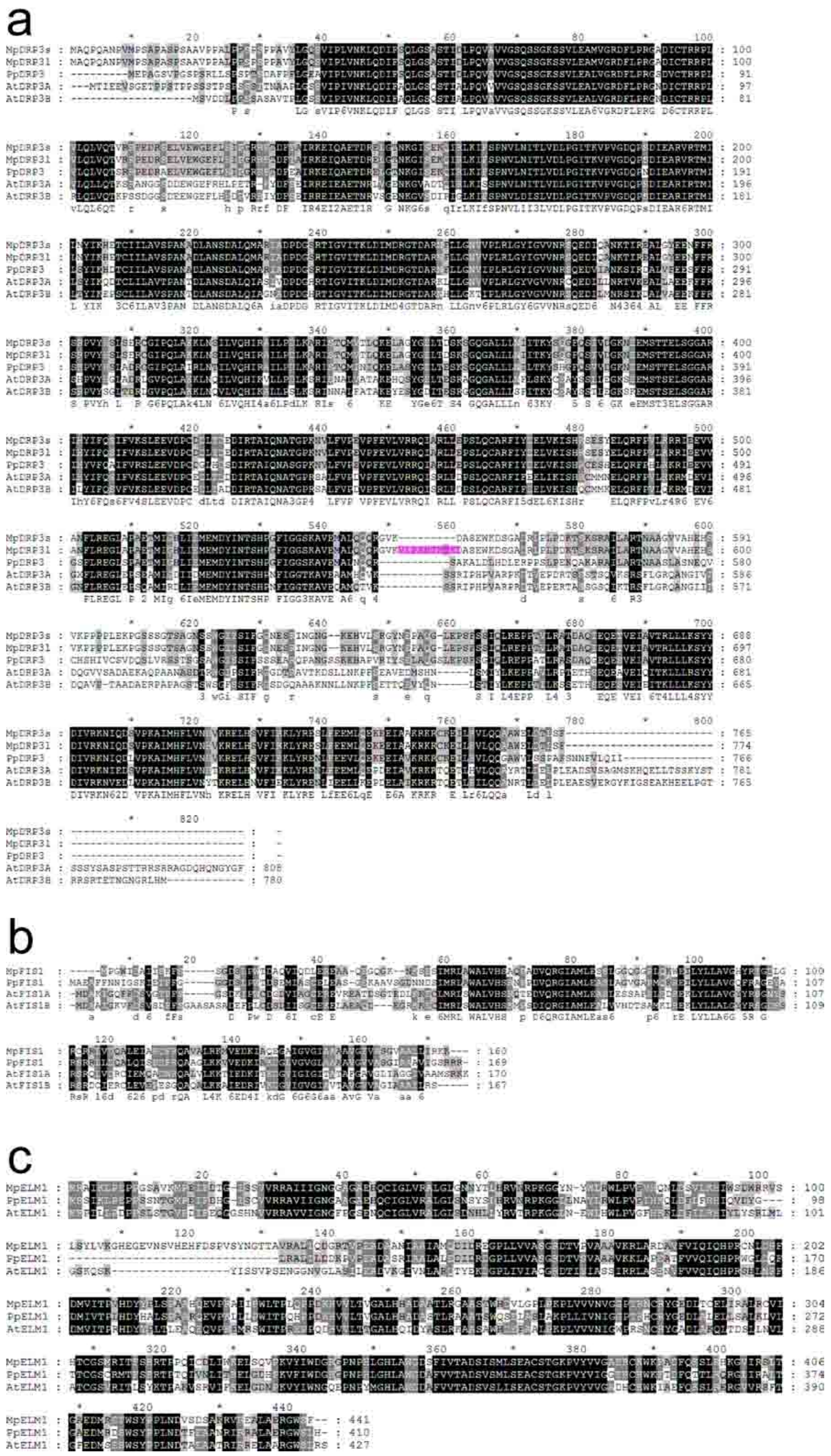
a
MpDRP3s
MpDRP3l
PpDRP3
AtDRP3A
AtDRP3B
b
MpFIS1
PpFIS1
AtFIS1A
AtFIS1B
c
MpELM1
PpELM1
AtELM1

Figure S1. Sequence alignments of Mp*DRP3*, Mp*FIS1* and Mp*ELM1*. (a) Amino acid sequence comparison of Marchantia, Physcomitrella and Arabidopsis DRP3 (MpDRP3s LC209090, MpDPR3l LC209090, PpDRP3 XM_001761482, AtDRP3A AB072374 and AtDRP3B AB072375). The magenta colored region indicates the predicted alternative splice site, a difference between MpDRP3s and MpDRP3l. (b) Amino acid sequence comparison of Marchantia, Physcomitrella and Arabidopsis FIS1 (MpFIS1 LC209092, PpFIS1 XM_001764394, AtFIS1A AB195717 and AtFIS1B AB195718). (c) Amino acid sequence comparison of Marchantia, Physcomitrella and Arabidopsis ELM1 (MpELM1 LC209091, PpELM1 XM_001766108 and AtELM1 AB379589).

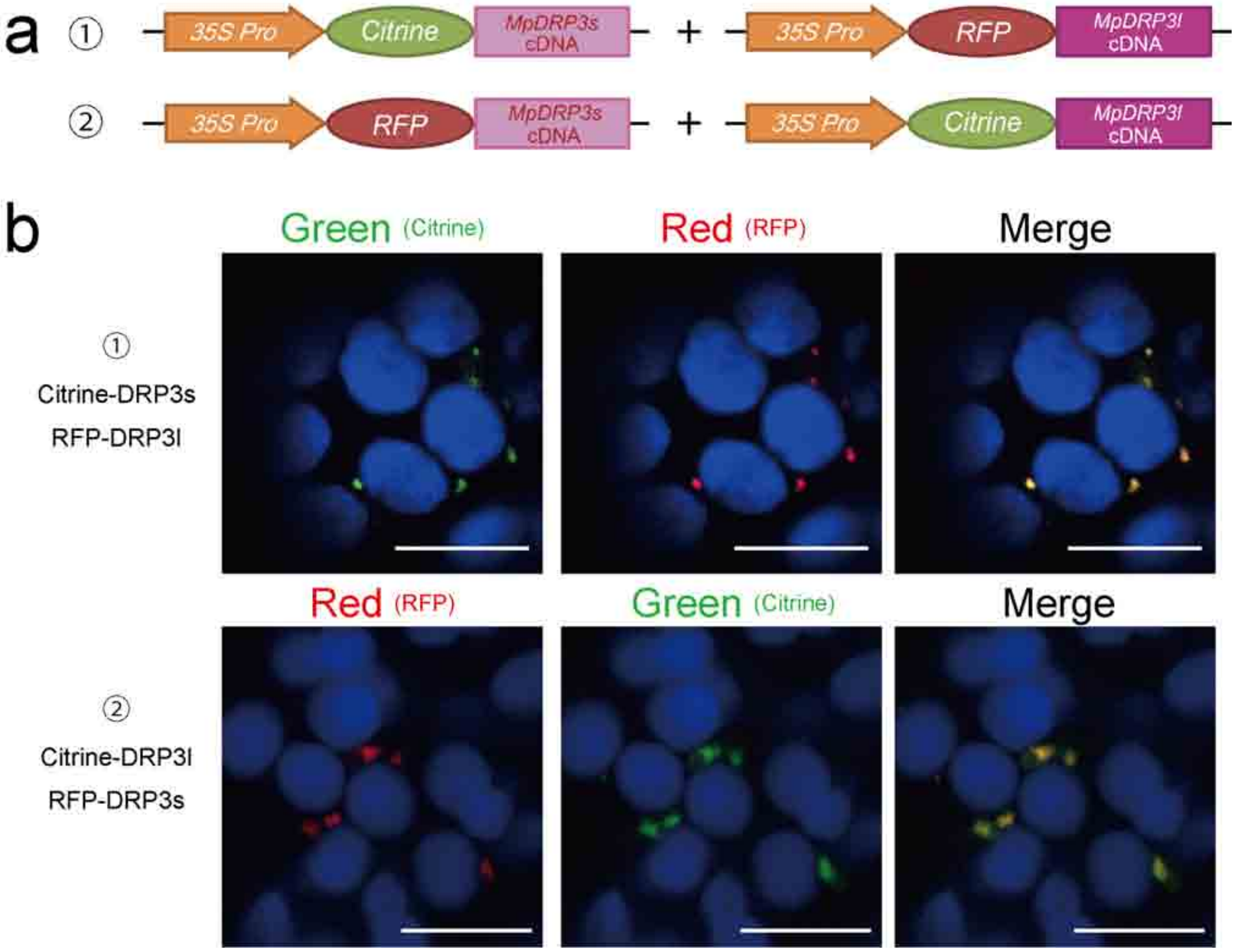

Figure S2. Microscopic observations of MpDRP3s and MpDRP3l simultaneously. (a) Schematic drawings of reporter gene constructions and the combinations. (b) CLSM images of the epidermal cells transiently transformed with the constructs by particle bombardment (Bars = 10 μm). Blue signals are chlorophyll autofluorescence.

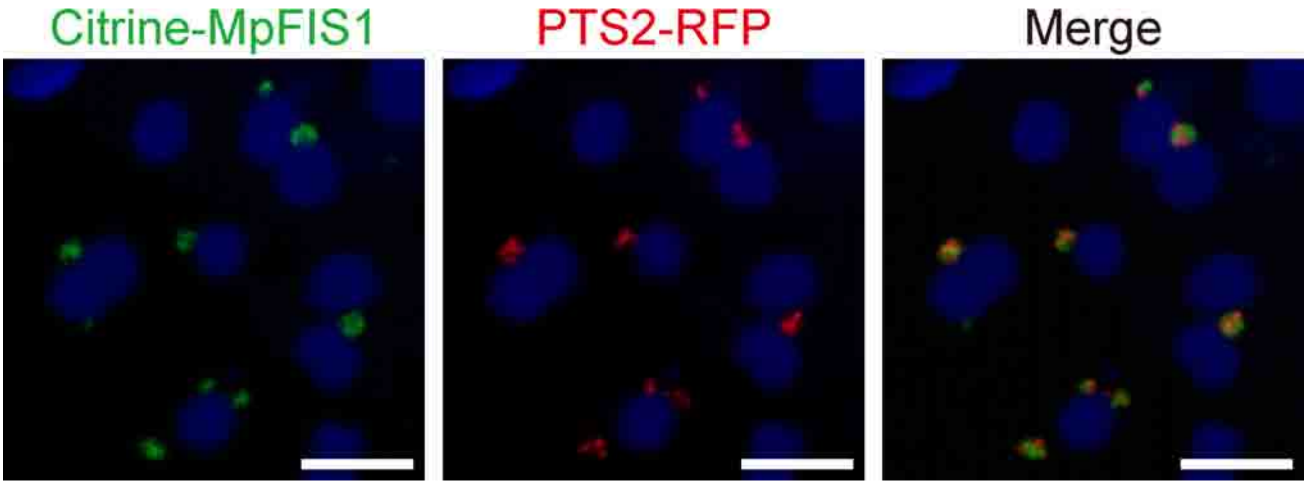

Figure S3. Microscopic observations of MpFIS1 and peroxisomes simultaneously. CLSM images of the epidermal cells transiently transformed with Citrine-MpFIS1 and PTS2-RFP by particle bombardment (Bars = 10 μm). Blue signals are chlorophyll autofluorescence. PTS2-RFP expression vector was kindly provided by Dr. Shoji Mano (Mano et al. (2006) Plant J. 47, 604-618).

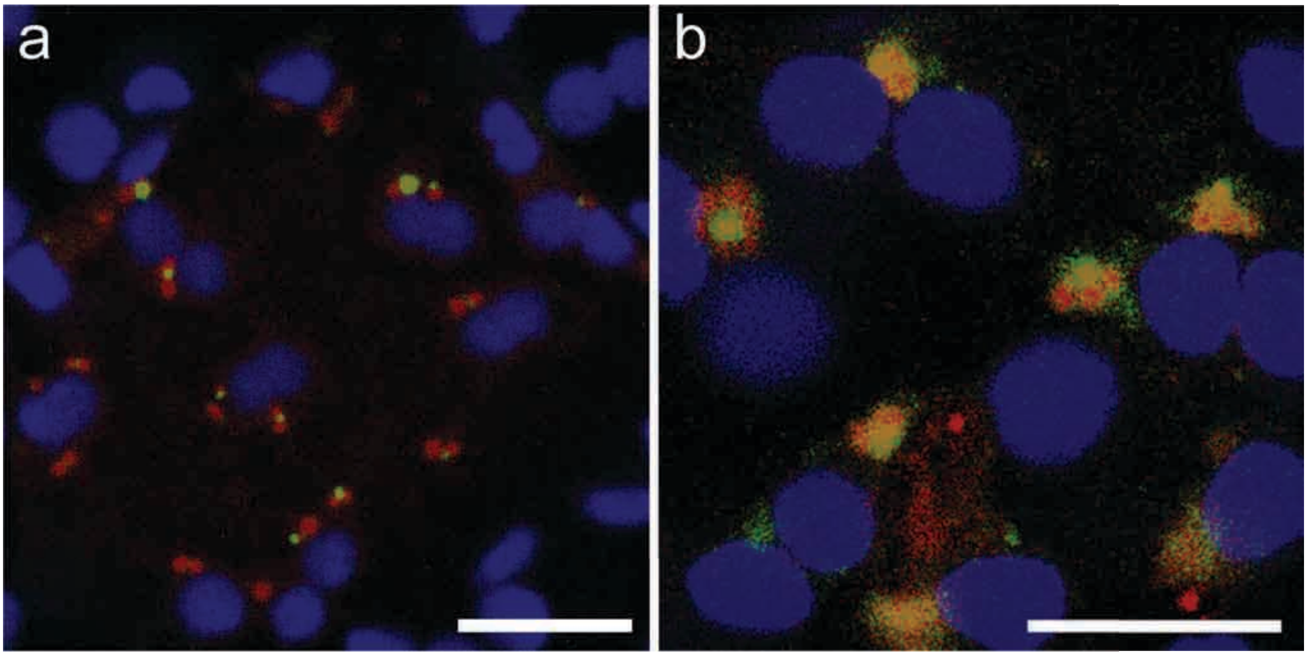

Figure S4. Microscopic observations of MpDRP3s and MpELM1 in Mp*fis1*. (a) CLSM images of the epidermal cells transiently transformed with Citrine-MpDRP3s and MtRFP in Mp*fis1* by particle bombardment. (b) CLSM images of the epidermal cells transiently transformed with MpELM1-Citrine and MtRFP in Mp*fis1* by particle bombardment (Bars = 10 μm). Blue signals are chlorophyll autofluorescence.

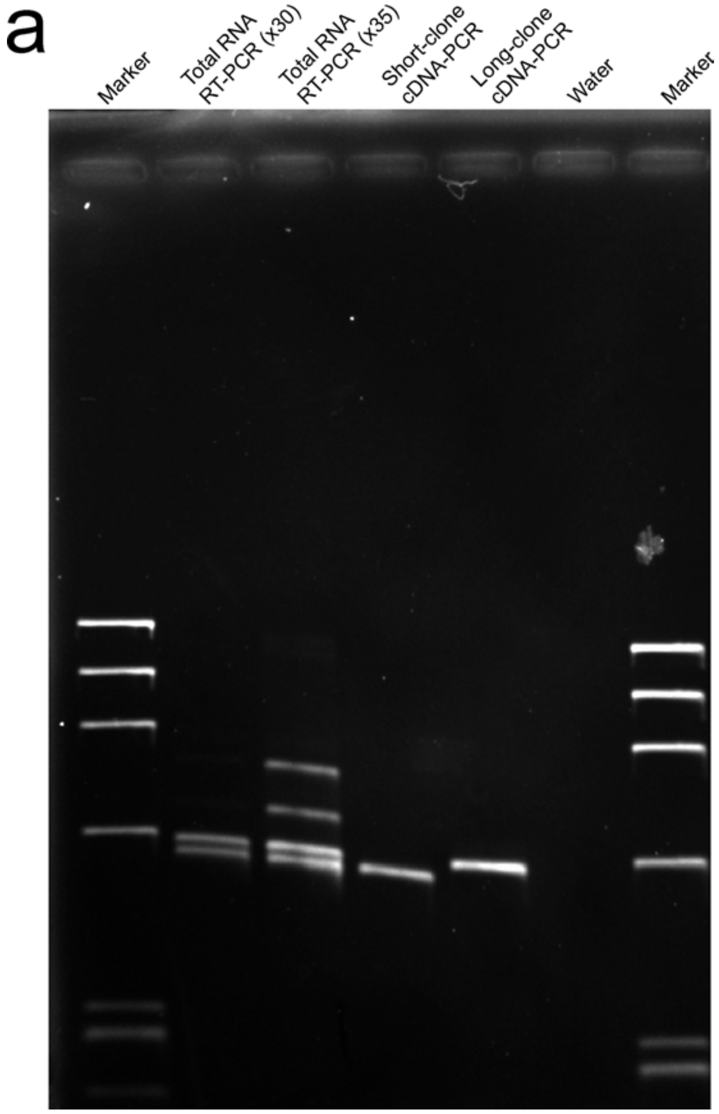

Figure S5. Display of full-length gel images of cropped gel images which appear in main text. (a) A full-length gel image of Figure 1b. Two major bands matched in size to the Mp*DRP3s* and Mp*DRP3l* are shown in Figure 1b and further analyzed in the paper. (b) Full-length gel images of Figure 3b.

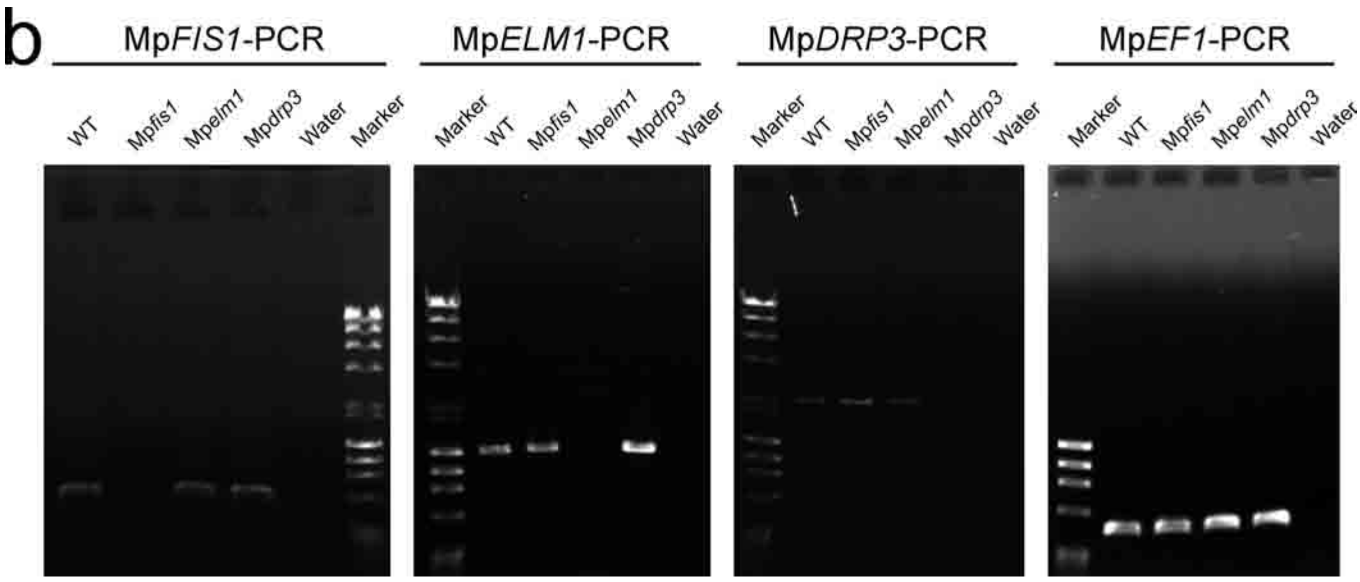

Table S1. Primers used in this study.

Name	Sequence (5'→3')	Usage
Primer 1	CACCATGCCTGGGTGGATTGA	For amplification of Mp*FIS1* cDNA
Primer 2	GGCAAACTACTTCTTGCGAATC	For amplification of Mp*FIS1* cDNA
Primer 3	CACCATGAGAGCCATAAAGCTGCCAGAA	For amplification of Mp*ELM1* cDNA
Primer 4	TTAAAAGCTCCAACCGCGCTC	For amplification of Mp*ELM1* cDNA
Primer 5	CACCATGGCGCAGCCTCAAG	For amplification of Mp*DRP3* cDNA
Primer 6	GAGATCAAAAAGACAAGGTGTCC	For amplification of Mp*DRP3* cDNA
Primer 7	CACCCCGTGCACTGATTGCTCCATTTGCATCCA	For amplification of Mp*DRP3* genomic DNA
Primer 8	AAAAGACAAGGTGTCCAGTTCCTGC	For amplification of Mp*DRP3* genomic DNA
Primer 9	GGACAGAGTTCCCGCGATTTT	For direct sequence of Mp*DRP3*
Primer 10	ACCAATTCCGAACGATCCTC	For direct sequence of Mp*DRP3*
Primer 11	GTAAAACGACGGCCAG	For sequence of Mp*FIS1* cDNA
Primer 12	CAGGAAACAGCTATGAC	For sequence of Mp*FIS1* cDNA
Primer 13	CGAGCCCTTACTCAAGATGG	For sequence of Mp*ELM1* cDNA
Primer 14	CACGGAGAACTCCTCCTCAG	For sequence of Mp*ELM1* cDNA
Primer 15	TGCCTGGCATTACAAAAGTG	For sequence of Mp*DRP3* cDNA
Primer 16	GAGGTGCCATTTGAGGTGTT	For sequence of Mp*DRP3* cDNA
Primer 17	GACACCAATCGTACGAGATCC	For sequence of Mp*DRP3* cDNA
Primer 18	GTTGATTCGCGACTCGTTTC	For sequence of Mp*DRP3* cDNA
Primer 19	CGCGGTTGGAGCTTTAAGGGTGGGCGCGCC	For site-directed mutagenesis of Mp*ELM1* cDNA
Primer 20	GGCGCGCCCACCCTTAAAGCTCCAACCGCG	For site-directed mutagenesis of Mp*ELM1* cDNA
Primer 21	TCACTCTGGGTGTGAAGCAG	For amplification of Mp*EF1*
Primer 22	GCCTCGAGTAAAGCTTCGTG	For amplification of Mp*EF1*
Primer 23	TGCGCCCAGGTAGCTTGGACCTCTTCAAAAGTGGG	For construction of Mp*FIS1* targeting vector
Primer 24	CTGTTATCCCTAGGCGAGTAACGCATCCGGAGAAT	For construction of Mp*FIS1* targeting vector
Primer 25	CTAAGGTAGCGATTAATACTACAGATGATGGCAGGAC	For construction of Mp*FIS1* targeting vector
Primer 26	CCGGGCAAGCTTTTAATCCTTTCAAGGTCCTGGAAGT	For construction of Mp*FIS1* targeting vector
Primer 27	TGCGCCCAGGTAGCTGATGCTGCTACTCTACGTGG	For construction of Mp*ELM1* targeting vector
Primer 28	CTGTTATCCCTAGGCCACTGCTACCAGAAACTGCT	For construction of Mp*ELM1* targeting vector
Primer 29	CTAAGGTAGCGATTAATGAATTCCCTATCGTGCTACC	For construction of Mp*ELM1* targeting vector
Primer 30	CCGGGCAAGCTTTTAATGGAATGATAGCTCGTGGAAC	For construction of Mp*ELM1* targeting vector
Primer 31	TGCGCCCAGGTAGCTCATCACACTCGTAGACTTGC	For construction of Mp*DRP3* targeting vector
Primer 32	CTGTTATCCCTAGGCCTCCCGACAACTCTGTTGTT	For construction of Mp*DRP3* targeting vector
Primer 33	CTAAGGTAGCGATTAATATCGTGACAACGCTCTAGAG	For construction of Mp*DRP3* targeting vector
Primer 34	CCGGGCAAGCTTTTAATCCTTGCGAATGGCGGTGAAA	For construction of Mp*DRP3* targeting vector

Table S2. Primer pairs for gene targeting.

Mutants	For amplification of *Asc* I sites		For amplification of *Pac* I sites	
	fw	rv	fw	rv
Mp*fis1*	Primer 23	Primer 24	Primer 25	Primer 26
Mp*elm1*	Primer 27	Primer 28	Primer 29	Primer 30
Mp*drp3*	Primer 31	Primer 32	Primer 33	Primer 34
